# Music literacy improves reading skills via bilateral orthographic development

**DOI:** 10.1038/s41598-024-54204-8

**Published:** 2024-02-12

**Authors:** Marta Maria Pantaleo, Giulia Arcuri, Mirella Manfredi, Alice Mado Proverbio

**Affiliations:** 1 Cognitive Electrophysiology Lab, Department of Psychology, University of Milano-Bicocca, Piazza Dell’Ateneo Nuovo 1, 20162 Milan, Italy; 2 Psychologisches Institut, University of Zurich, Zurich, Switzerland; 3 Milan Center for Neuroscience, NeuroMI, Milan, Italy

**Keywords:** Reading, Musicians, ERPs, N170, VWFA, Hemispheric asymmetry, Neuroplasticity, Dyslexia, Neuroscience, Psychology, Neurology

## Abstract

Considerable evidence suggests that musical education induces structural and functional neuroplasticity in the brain. This study aimed to explore the potential impact of such changes on word-reading proficiency. We investigated whether musical training promotes the development of uncharted orthographic regions in the right hemisphere leading to better reading abilities. A total of 60 healthy, right-handed culturally matched professional musicians and controls took part in this research. They were categorised as normo-typical readers based on their reading speed (syl/sec) and subdivided into two groups of relatively good and poor readers. High density EEG/ERPs were recorded while participants engaged in a note or letter detection task. Musicians were more fluent in word, non-word and text reading tests, and faster in detecting both notes and words. They also exhibited greater N170 and P300 responses, and target-non target differences for words than controls. Similarly, good readers showed larger N170 and P300 responses than poor readers. Increased reading skills were associated to a bilateral activation of the occipito/temporal cortex, during music and word reading. Source reconstruction also showed a reduced activation of the left fusiform gyrus, and of areas devoted to attentional/ocular shifting in poor vs. good readers, and in controls vs. musicians. Data suggest that music literacy acquired early in time can shape reading circuits by promoting the specialization of a right-sided reading area, whose activity was here associated with enhanced reading proficiency. In conclusion, music literacy induces measurable neuroplastic changes in the left and right OT cortex responsible for improved word reading ability.

## Introduction

Professional musicians' brains constitute a model of experience-dependent brain plasticity that has attracted increasing interest in neuroscience^1–6^. Several brain areas, including the anterior corpus callosum, cerebellum, primary motor area and auditory cortices, show differences in structure and size in musicians compared to non-musicians^3^. Higher demands for bimanual coordination and rapid information exchange appear to promote neural fiber growth^7^, or at least prevent neural tissue loss during synaptic pruning^8^. In particular, the volume of the cerebellum is larger in musicians than in non-musicians^2,9,10^ because of its role in regulating coordination, precise timing and accuracy of motor commands. Other structural and functional changes due to musical experience have been observed in somatosensory and motor cortex^11–14^, and along the auditory pathway: in the brainstem^15^, primary auditory cortex^16^, and higher-order auditory areas^17,18^.

These neuroplastic changes are associated with the multiple benefits of music education in terms of improvements in attentional, executive, coordination, motor, cognitive, and motivational skills (e.g.,^19–21^). Specifically for reading, the benefit would come from refined auditory processing and increased phoneme processing, rhythm and phonological awareness skills (e.g.,^22,23^) on the one hand, and improved ability to visually decode symbols and quickly translate them into meanings and gestures on the other. Several studies have shown how musical perceptual skills correlate with phonological awareness^24^ and reading skills, and may also be predictive of children's reading developmental trajectories^25–28^. For example, an interesting meta-analysis^29^ showed that students who received music education scored significantly higher on reading tests than control students. Again, Standley and Hughes^30^ and Register^31^ gave music lessons to 4–5 year old children and compared them with a control group of children of the same age who did not receive music lessons, and showed that music lessons at an early age contributed to improvements in pre-reading and writing skills. Swaminathan et al.^32^ observed a large group of 166 native and non-native English-speaking adults who had an average of two years of private music lessons outside of school and found a positive correlation between music practice and reading skills, as also found by Schellenberg and Weiss^21^.

Despite this body of knowledge, not much is known about the plastic changes in the brain that underlie improved reading skills, particularly with regard to the visual encoding of orthographic and symbolic information such as words and musical notation. Here, we tested the hypothesis that musical literacy (the ability to read music fluently) acquired at the age of 10 or earlier, and continued practice of this ability, would have a direct impact on the ability to read letters and words. This would occur through the development of a right hemispheric reading area (used for pentagram reading). In addition, the intensive training of attention and eye shifting involving V5 and the oculomotor area^33^ would also act as an enhancing and protective factor for reading ability.

A previous ERP study by Proverbio et al.^34^ compared the ability to identify notes and letters (embedded in musical bars and words, respectively) in groups of musicians and matched controls. The results showed that the orthographic N170 was larger for words in musicians than in controls. In addition, the N170 response showed a bilateral distribution in musicians during the processing of both notes and words, whereas it was strongly left-sided in controls, as expected. Source reconstruction showed that the fusiform (BA37) and inferior occipital gyri (BA18) were activated in both hemispheres in musicians (for both word and music processing), whereas orthographic processing was restricted to the left hemisphere in controls. This neural pattern was associated with an enhanced ability to recognise both notes and letters in musicians compared to controls. We concluded that the neural mechanisms of word reading can be modified by musical training in childhood (from the age of ~ 8 years). The evidence of right hemisphere involvement for a function normally lateralised to the left seems to be a consequence of the neuroplastic effects of musical training on reading ability. This hypothesis is supported by the findings reported by Li and Hsiao^35^, who investigated the effects of musical reading on reading in an English word and Chinese character naming task presented in different visual fields. They found an effect of musical literacy on word reading lateralization: in fact, musicians performed significantly faster than controls for words presented in the left visual field (right hemisphere). The literature consistently demonstrates that bilateral neural mechanisms, primarily relying on the right OT cortex, underlie the ability to read musical notation. In different paradigms, the so-called *Visual Note From Area* (VNFA) has been localised in the right transverse occipital sulcus, right occipital gyrus, right inferior occipital gyrus, right occipitotemporal junction, right fusiform gyrus and right superior parietal cortex^36,37^ and supramarginal cortices^34,38–45^. If music education and word reading skills are linked, and if the former is able to influence the development and performance of reading skills, then it can be expected that treatment based on learning music skills might contribute to the improvement of language skills in individuals who are deficient in these skills. Indeed, Register et al.^46^ and Habib et al.^47^ investigated whether music could be a strategy to improve reading skills in students with specific reading difficulties and found significant improvements in reading tests administered before and after music training.

If it were true that the development of a right-sided orthographic area (necessary for encoding spatial relations in pentagrams, as originally reported by Proverbio et al.^34^ was the cause of the better performance of musicians in orthographic tasks and reading, then we would expect not only a bilateral activation of orthographic areas in musicians (during word reading), but also their better performance in independent reading tests. In addition, the *Visual Word Form Area* (VWFA) should show less activation in poor readers than in good readers. The VWFA, located in the medial part of the left fusiform gyrus, is known to underlie the ability to recognise letters and words, being more sensitive to letter strings than to other pictorial stimuli^48–53^, while also being sensitive to sub-lexical properties such as word familiarity or frequency of use^54–56^. The development and specialisation of this area for the recognition of written words enables rapid reading by increasing the perceptual capacity for words and making it sensitive to recurrent features of the writing system^57^. In this respect, the N170 component of ERP is deemed as the electromagnetic representation of VWFA activity. Research has proven that the amplitude of the N170 response is higher when presented with letters or words instead of other objects. Moreover, this response is typically focused over the left occipito/temporal area^55,58^.

The present ERP study aimed to investigate the pattern of brain electrical activity in a sample of professional musicians and controls in response to words and musical notation. Right hemispheric involvement in word reading (in addition to music reading) was expected in areas contralateral to the VWFA in musicians, as previously demonstrated by Proverbio et al.^34^. In the present study, a parallel comparison was made between good and poor readers within the large sample of participants. We hypothesised: (1) that professional musicians have developed a specialised visual area over the right OT for reading music notation, the activity of which is reflected in a bilateral N170 response to notes (obviously larger than in controls); (2) that musicians also automatically activate the bilateral orthographic areas for word processing (whereas the N170 is purely left-sided in non-musicians); (3) that musicians were better readers than controls and showed larger amplitudes of N170 and P300 components; that good readers, regardless of musical ability, showed bilateral involvement of orthographic areas; (4) that N170 amplitudes correlated with reading ability, thus proving to be a reliable marker of reading ability. It is important to note that in order to prevent transfer of perceptual strategies across tasks, the musical notation and word experimental sessions were independent and separated in time (in different experimental blocks).

Few studies have previously compared N170 and P300 potentials elicited by written words (reflecting orthographic and selective attention processes, respectively) in musicians vs. non-musicians. Proverbio and co-authors^34^ found larger N170 and N250 responses (*selection negativity*) to written words in musicians compared to control readers, as well as faster response times (RTs) and higher accuracy in letter identification tasks, but no larger P300 responses. Li and coauthors^35,44^ found earlier RTs, higher accuracy and larger N170 responses (also over the right hemisphere) in musicians, in word naming tasks, but did not quantify P300 responses. Overall, there is no consolidated evidence that the P300 is larger in musicians during visual reading tasks, whereas it is commonly reported that the P300 is larger in musicians compared to non-musicians in auditory or speech listening tasks (e.g.^59^) due to neuroplastic changes in the auditory cortex.

## Material and methods

### Participants

Sixty right-handed, age-matched, healthy students took part in this study. They were half male and half female, matched for age and socio-cultural status, and all native Italian speakers. Specifically, the musicians were 34 individuals (17 females) aged 18–28 years (22.8; SD = 2.68). Inclusion criteria were being a professional musician, having graduated (BA or Master) from a music conservatory in the Lombardy region. They specialised in a variety of instruments: piano, guitar, opera singing, saxophone, oboe, violin, electric bass, bassoon, double bass, euphonium, transverse flute, organ, cello, trumpet and composition. Their average number of years of music study was 12.5 years (SD = 4.26), with an average age of acquisition (AoA) of 10 years, confirming that the musicians were truly experienced and professional. Four musicians were excluded due to excessive EEG artifacts. The final sample for ERP analyses consisted of 30 musicians (15 females) aged between 18 and 28 years (22.7; SD = 2.64). Their lateral preference was assessed by administering the Edinburgh Inventory. The musicians' mean laterality score was 0.75 (mean = 0.7479, min = 0.4286, max = 1.0, SD = 1.18).

Participants were preliminarily interviewed about their reading habits and musicians self-reported an average number of printed pages read (per year) = 1250, equivalent to approximately 3.5 books (including college textbooks). Controls were 26 graduated (BA or master) University student of non-musical faculties in the Lombardy area (21 females). They aged 19–27 years (22.12; SD = 1.82); their average laterality score was 0.80 (mean = 0.7999, min = 0.4300, max = 1.0, SD = 1.145). No control participant was discarded because of EEG artifacts. Non-musicians reported an average number of printed pages read (in a year) = 2915, equivalent to about 8 books (including college textbooks). An ANOVA performed on the laterality scores showed no effect of group (*p* = 0.26). A further ANOVA on the number of pages read in 1 year showed a significant effect of group (*p* < 0.0002), as expected. Inclusion criteria for the control group were: never having studied music (except for a few years in junior high school), not currently able to read music, not playing or singing, and not studying a musical instrument as a hobby.

Inclusion criteria for all 60 participants were that they had never had a psychiatric or neurological disease or injury, were not currently taking drugs or narcotics, and were not predisposed to epilepsy. All participants had (self-reported) normal or corrected-to-normal vision and hearing. No participant suffered or had ever suffered from a learning or reading disorder (e.g. developmental dyslexia, alexia, autism, ADHD, etc.). All participants gave written informed consent before taking part in the study and were unaware of the specific purpose of the research. The experiment lasted approximately 3 h and participants volunteered or received academic credit. All methods were performed in accordance with the relevant guidelines and regulations (Declaration of Helsinki). The project was approved by the Ethics Committee of the University of Milan-Bicocca (protocol number RM-2021-370).

### Reading tests and questionnaires

The study consisted of two experimental sessions, separated by a few days to reduce subject fatigue: the first session consisted of the assessment of inclusion criteria, the collection of demographic data and the administration of the reading and laterality tests; the second session consisted of the recording of EGG/ERP signals. Subjects were recruited through announcements on the university's social networks. Volunteers first provided some preliminary information, such as age, degree, eventual academic level in music attained, age of acquiring the ability to play an instrument, primary instrument, secondary instrument, and identification of presumed right-hand preference. After recruitment, both musicians and controls participated in an initial meeting via a digital platform, and the video was recorded for data analysis with the participant's consent.

In this session, a reading battery (in Italian) was administered, consisting of word and nonword reading lists from the *Battery for the Assessment of Developmental Dyslexia and Dysorthographia*^60^ and a text from the *VALS-Assessment of Reading and Writing Difficulties in Adulthood*^61^. The word reading test consisted of 4 runs of vertically ordered familiar words (28 words per set), administered individually. The nonword reading test consisted of 3 sets of 16 pronounceable nonwords. The text reading consisted of a one-page narrative (taken from Stefano Benni's novel "The Bar Under the Sea") that had to be read quickly but with appropriate prosody. The order in which the tests were administered was randomised across subjects. Reading accuracy and speed were calculated for each test (word, pseudoword and text reading). Participants were asked to read the text displayed on the screen as quickly as possible, but as accurately and clearly as possible. Five warning signals preceded the start of the test. "Attention", "Three, Two, One", "Go". Reading times were calculated from the start of the participant's reading to the end of vocal production. After each performance, all participants received the same general positive reinforcement and thank you. Following the administration of the reading tests, participants were administered the Edinburgh Inventory Questionnaire to assess their lateral preference.

### Stimuli and procedure

**Figure 1:**
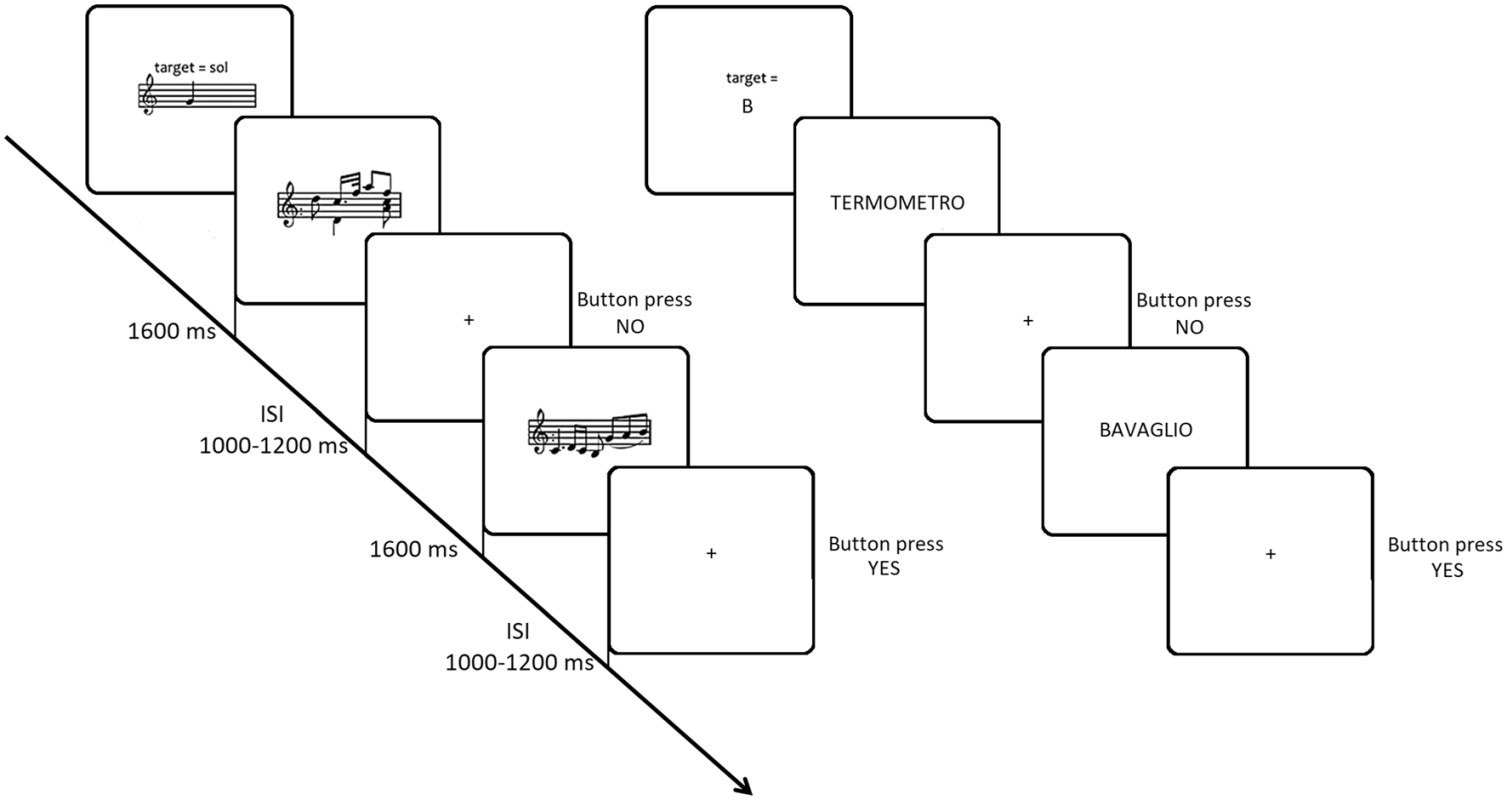
Time sketch of experimental procedure for the music and language conditions.

The stimuli consisted of 300 Italian words and 300 music bars of different length and complexity, presented in random order in the centre of a PC screen, approximately 114 cm from the subject's eyes (Fig. 1). The stimuli and procedure were the same as those used in the ERP study by Proverbio et al.^34^.

Words were typed in capitals in Arial Narrow and were 0° 30′ 11″ (1 cm) high and from 1° 15′ 27″ to 4° 31′ 37″ (2.5 to 9 cm) long. Music bars were 0° 45′ 16″ (1.5 cm) high and 4° 16′ 32″ (8.5 cm) long. Music bars varied in length from 4 to 8 notes, while words varied in length from 4 to 10 letters. Music fragments were selected from Mozart and Schumann real pieces for piano and violin. Two different experimental conditions were used: a note recognition task and a letter recognition task. Half of the participants completed the music task in the first half of the experiment and the orthographic task in the second half, while the order was reversed for the other half of the participants. In the orthographic task, 300 Italian words (half target, half non-target) were presented pseudorandomly in the centre of the screen. Stimuli lasted 1600 ms and the ISI ranged from 1000 to 1200 ms. All stimuli were matched for duration across trials and across target and non-target categories. Words were also matched for frequency of use (across target/non-target categories). Word frequencies were taken from a large online database of Italian words (*ColFIS*^62^).

The stimuli used as targets were 'mi', 'fa', 'sol', 'la' and 'si' of the middle piano octave (i.e. E4, F4, G4, A4 and B4) for the note recognition task and the letters B, G, L, M and S for the orthographic recognition task. At the beginning of each session, subjects were told what the targets were and which hand would be used to respond. Subjects were given both a verbal label and an enlarged visual representation of the isolated letter or note (within the pentagram), which remained in the subject's hand for free inspection until the experimental trial began. Participants sat comfortably in an acoustically and electrically shielded booth under scotopic luminance conditions in front of a high-resolution monitor placed outside the booth. They were asked to keep their gaze fixed on a 3 mm fixation point at the centre of the screen, minimising eye movements and avoiding any body movements. The task was to respond as quickly and accurately as possible by pressing a button on a joystick with their index finger each time they detected the target within the stimulus. The experimenter indicated the response hand, which alternated between runs, before the start of each sequence. Stimuli were presented in 12 runs of 50 stimuli each, half target and half non-target, randomly intermixed. The order of response hands was counterbalanced across participants, and the order of presentation of word and tone sequences was also randomised and counterbalanced. The two experimental sessions were preceded by two training sequences using exemplar stimuli that would not be repeated later in the experiment. Each sequence began with the appearance of the words 'Ready', 'Attention' and 'Go' written in block letters and ended with the words 'Thank you'.

### EEG recordings and data analysis

EEG data were recorded using a standard 128-electrode EEG cap placed according to the 10–5 international system^63^ with *EEProbe* v2.2 software (ANT Neuro, Hengelo, The Netherlands) at a sampling rate of 512 Hz (bandpass 0.16–70 Hz). Horizontal (hEOG) and vertical (vEOG) eye movements were also recorded. Linked mastoids were used as reference lead. Electrode impedance was kept below 5 KOhm. Computerised artefact rejection and manual eye inspection were used to remove EEG segments contaminated by eye artefacts (saccades and blinks), muscle-related potentials or amplifier blockages. The computerised criterion for rejecting artefacts was a peak-to-peak amplitude exceeding 50 μV. EEG epochs were synchronised with stimulus onset. Evoked response potentials (ERPs) were averaged off-line from 100 ms before to 1500 ms after stimulus onset, and an off-line filter (band-pass 0.16–30 Hz) was applied to the ERPs. Data from reading tests, behavioural responses and EEG/ERP recordings were analysed.

The results of the reading tests were normed following the protocols of the battery for the assessment of dyslexics and dysgraphias^60^ and the VALS test^61^. The sample was divided into 'good readers' and 'poor readers' groups based on their average reading speed (number of syllables read per second), both within each group (musicians vs. controls) and considering the whole population. Correlation analyses were performed between the scores on the three reading tests, between word reading speed (syllables per second = reading ability) and TRs to target words, and between reading ability and the amplitude of N170 and P300 responses to target words.

ERP components were identified in the time window and scalp location where and when they reached maximum amplitude and according to previous literature. The mean area amplitude of the N170 was quantified at occipito-temporal sites (PPO9h-PPO10h). The N1 peak was defined as the most negative value between 170 and 210 ms. The mean area amplitude P300 was quantified in the 600–800 ms time window for notes and in the 450–650 ms time window for words at centro/parietal sites (CP1-CP2).

For each ERP component, repeated-measures ANOVAs were applied to individual ERP amplitudes recorded in musicians and controls, as a function of reading proficiency (poor vs. good readers), and stimulus type. In details, between-group factors were: Proficiency (poor vs. good readers) and Group (musicians vs. controls). Within-group factors were: stimulus type (notes vs. words), attention (non-targets, targets), hemisphere (left, right).

Further repeated-measures ANOVAs were performed on response times (RTs) and accuracy data. Between-group factors were: proficiency and Group (Musicians vs. Controls). Within-group factors were: stimulus type (note vs. word), targetness (non-target, target) and response hand (left, right).

Tukey and Fisher post-hoc comparisons were carried out to test differences among means. The effect size for the statistically significant factors was estimated using partial etasquared (η_p_^2^) and the Greenhouse–Geisser correction was applied to account for non-sphericity of the data. All the ANOVAs were performed using *Statistica* software (version 10) by StatSoft.

Spearman Rho correlation analyses were performed between individual values of N170 recorded in response to words in the left and right hemispheres and proficiency as measured by syllable/sec reading speed. Furthermore, response times were correlated with the amplitude of the P300 response to target words as recorded at centro/parietal CP1-CP2 electrode pair. Correlation analyses also performed to assess the relationship between reading performances on the three reading scales.

### Source reconstruction

Low-resolution electromagnetic tomography (LORETA) was performed on ERP waveforms at the N170 latency (170–210 ms) during selective attention to letters or notes in good and poor readers, musicians and controls. Further LORETAs were performed on ERP waveforms at the P300 latency (450–650 ms) during selective attention to letters, in musicians and controls. LORETA is an algorithm that provides discrete linear solutions to inverse EEG problems. The resulting solutions correspond to the 3D distribution of neuronal electrical activity that has the most similar orientation and strength between neighbouring neuronal populations (represented by adjacent voxels). This study used an improved version of this algorithm, the standardised weighted (sw)LORETA^64^. This version, referred to as swLORETA, incorporates a singular value decomposition based source field weighting method. The source space properties included a grid spacing (the distance between two computation points) of five points (mm) and an estimated signal-to-noise ratio, which defines the regularisation, with a higher value indicating less regularisation and therefore less blurring of the results, of three. Using a value of 3–4 for the calculation of SNR in Tikhonov's regularisation results in superior accuracy of solutions for each inverse problem evaluated. swLORETA was run on the grand-averaged group data to identify statistically significant electromagnetic dipoles (*p* < 0.05) where larger magnitudes correlated with more significant activation. As part of the LORETA analysis, the data were automatically referenced to the mean reference. A realistic boundary element model (BEM) was derived from a T1-weighted 3D MRI dataset. This was achieved by segmenting the brain tissue. This BEM model consisted of a homogeneous compartment with 3,446 vertices and 6,888 triangles. Advanced Source Analysis (ASA) uses a realistic head model consisting of three layers (scalp, skull and brain) and is generated using the BEM. This realistic head model consists of a set of irregularly shaped boundaries and the conductivity values for the compartments between them^65^. A number of points connected by planar triangles are used to approximate each boundary. The triangulation results in a more or less evenly distributed mesh of triangles, depending on the chosen grid spacing. A smaller value for the grid spacing results in a finer mesh and vice versa. For the three-layer realistic head model mentioned above, it is assumed that the segmentation includes current generators of brain volume, including both grey and white matter. The regional conductivities of the scalp, skull and brain were assumed to be 0.33, 0.0042 and 0.33 respectively. Source reconstruction solutions provided by the Montreal Neurological Institute were projected onto the 3D MRI of the Collins brain. SwLORETA was performed on the grand mean group data to identify statistically significant electromagnetic dipoles (*p* < 0.05), where larger magnitudes correlated with more significant activations. Probabilities of source activation based on Fisher's F-test were provided for each independent EEG source, with values reported on a "unit" scale in nA (the larger the value, the more significant). Different colours indicate different strengths of electromagnetic signals. Both segmentation and head model generation were performed using *ASA* software (*ANT*, Enschede, The Netherlands). Topographic distributions of surface voltage were generated by mapping isopotential lines, derived by interpolating voltage values between surface electrode sites at specific time latencies, onto a colourimetric scale.

## Results

### Reading tests

Table 1 shows the results of the three reading tests for musicians and controls. The participants were divided into two subgroups, 'poor readers' (1st to 50th percentile) and 'good readers' (51st to 100th percentile), on the basis of the performance obtained, and in particular the speed of reading syllables per second for words. It is important to note that the participants did not suffer from dyslexia or alexia, nor did they have any reading or learning disorder; they were normative readers, as evidenced by their performance (see Table 1).

**Table 1:** Reading performance, expressed in terms of mean number of syllables per second (in word, non-word and text reading), and recorded for musicians (top rows) and controls (bottom rows) according to whether they were good or poor readers. Participants were ranked based on word reading time (from fastest to slowest) and, for simplicity, divided into the highest and lowest subgroups within their category. Reading speed was calculated by dividing the number of syllables read by the time (in seconds) taken to read them.

Proficiency	Words		Non_words		Text	
	Syl/Sec	#Errors	Syl/Sec	#Errors	Syl/Sec	#Errors
Musicians						
Good (N = 15)	6.74	0.46	4.14	1.49	7.60	10.40
Poor (N = 15)	5.14	0.56	3.47	1.84	6.20	14.40
Mean	**5.94**	0.51	**3.805**	1.67	**6.9**	12.4
Controls						
Good (N = 13)	6.28	0.48	4.10	1.69	7.17	10.69
Poor (N = 13)	4.78	0.37	3.05	1.56	6.08	10.77
Mean	**5.53**	0.42	**3.375**	1.62	**6.625**	10.73
Whole sample (normotypical readers)						
Group Mean	5.735	0.465	3.69	1.64	6.762	11.56
Normative data, 1st year University students (Re et al., 2011)	**5.14**	–	**3.21**	–	**5.96**	–

Average reading speeds (in syllables per second) for the group of musicians and controls (regardless of reading ability) and normative values for first-year university students are shown in bold.

The negligible number of errors made when reading words or non-words confirms that all participants were healthy, normo-typical readers. As slowing down, uncertainty or repetition were also considered errors, a few errors were observed when reading the whole page of an unfamiliar text with the constraint of being very fast. The slightly higher number of reading inaccuracies for text reading among musicians may be related to the significantly lower number of book pages per year that they self-reported. In addition, the slightly higher fluency of the controls compared to the normative data (especially in text reading) could be due to their cultural status (being university graduates rather than first year students).

**Figure 2:**
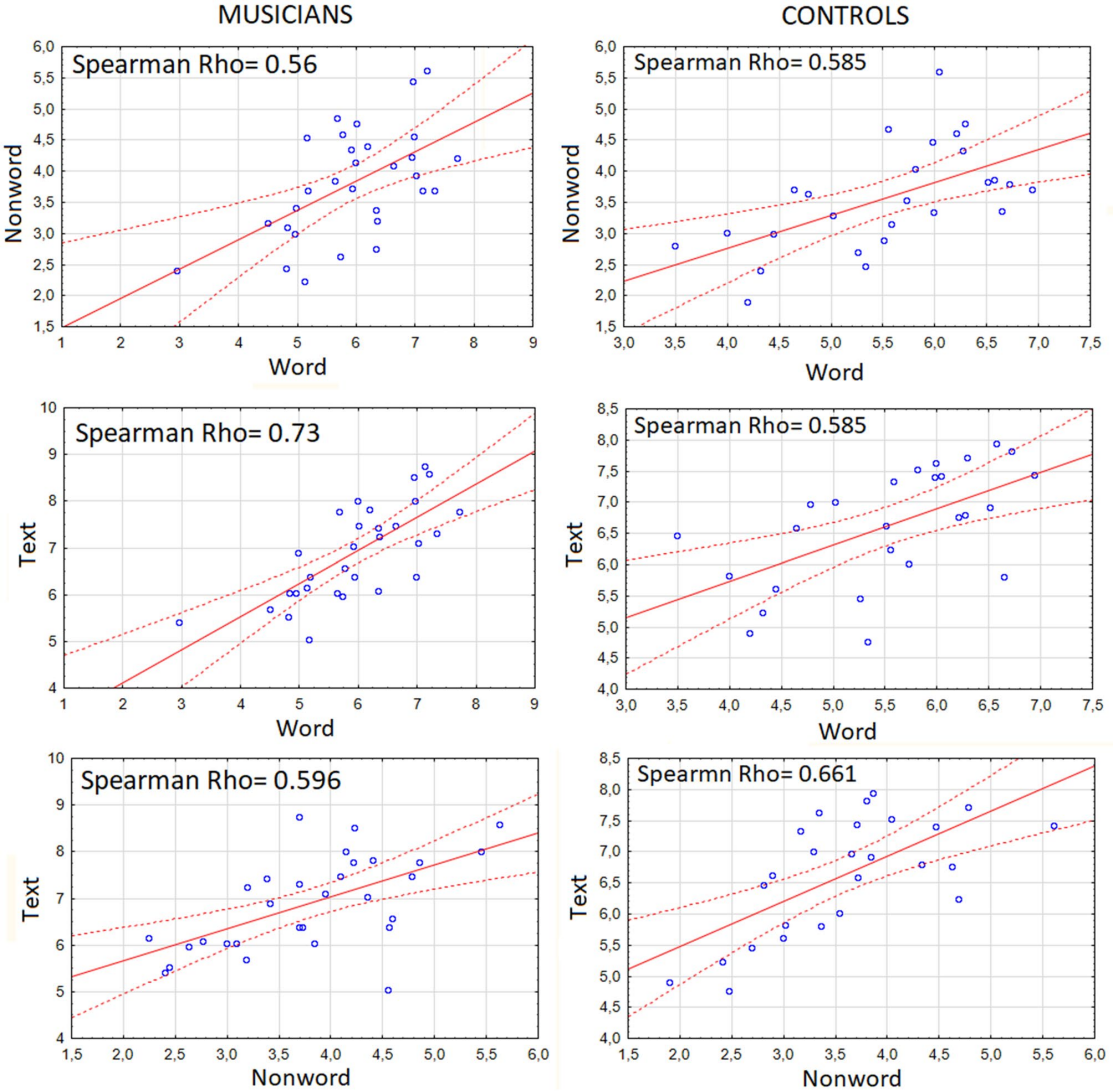
Correlation matrices displaying the relationship between the reading performances acquired in the three reading tests: word reading, nonword reading, and text reading in musicians (left column) and controls (right column).

Correlation analyses performed to assess the relationship between performance on the three scales showed a significant correlation between: word reading and non-word reading (r = 0.56, *p* < 0.05); between word reading and text reading (r = 0.73, *p* < 0.05); between non-word reading and text reading (r = 0.60, *p* < 0.05) (Fig. 2, left), indicating the reliability of the tests.

The lower part of Table 1 shows the results of the three reading tests for the controls (non-musicians). On the basis of the performance obtained, and in particular the speed of reading syllables per second for words, the participants were divided into two subgroups of "poor readers" (1st to 50th percentile) and "good readers" (51st to 100th percentile). Correlation analyses performed to assess the relationship between performance on the three scales showed a significant correlation between: word and non-word reading (r = 0.58, *p* < 0.05); word and text reading (r = 0.58, *p* < 0.05); non-word and text reading (r = 0.66, *p* < 0.05) (Fig. 2, right).

**Figure 3:**
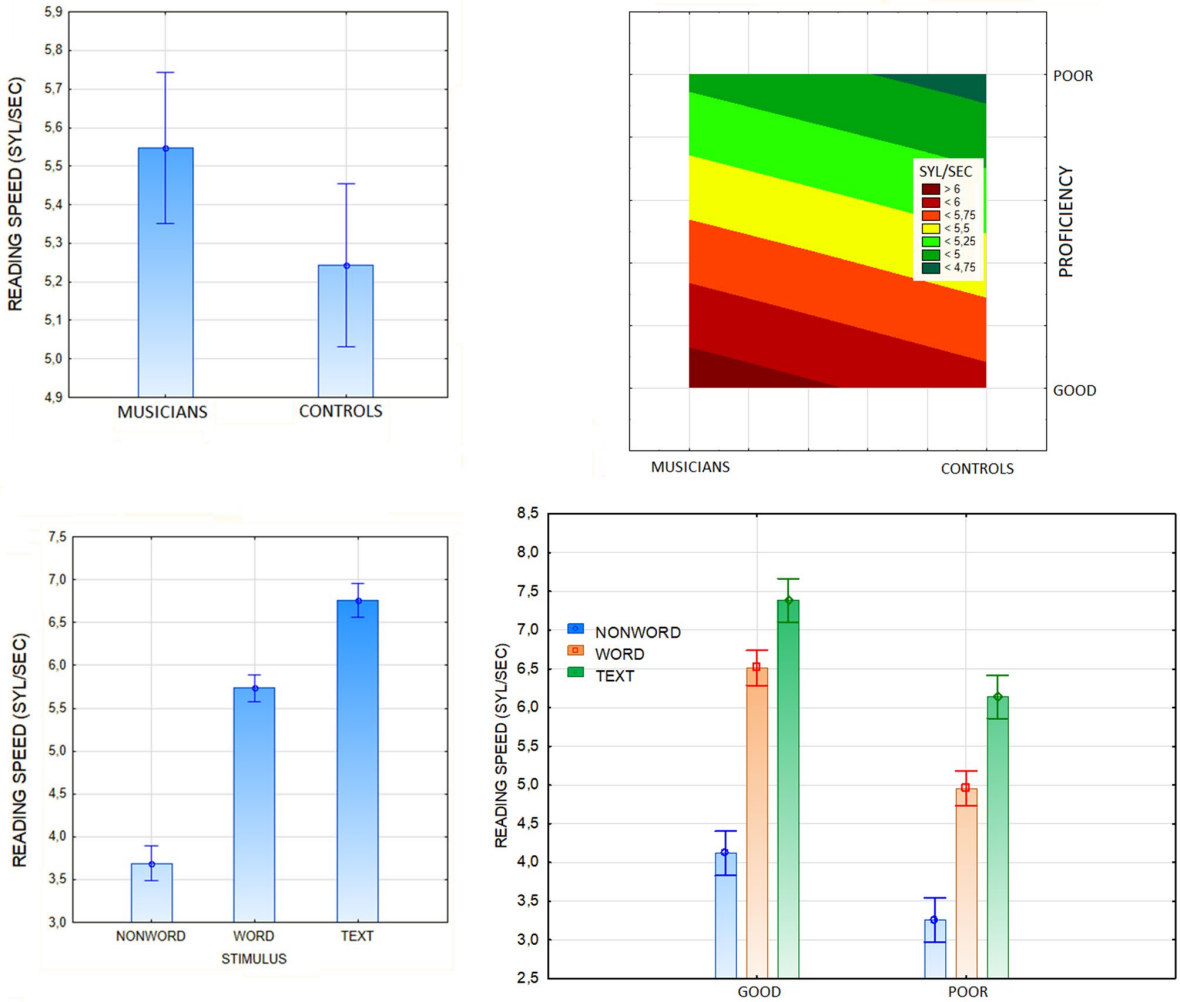
The data for reading tests are presented here, indicating reading speed for musicians and controls in comparison, for both good and poor readers. Additionally, reading speed (in syllables per second) is plotted as a function of stimulus type (word, non-word or text), and reading speed is compared between good and poor readers for each stimulus type.

An ANOVA performed on the whole sample to compare reading speed in the two subgroups revealed the strong effect of musicianship [F(1, 52) = 4.5236, *p* = 0.038], with musicians reading faster than controls regardless of proficiency (Fig. 3). The significance of the proficiency factor [F(1, 52) = 72.7, *p* < 0.00001] indicated faster reading times for good readers than for poor readers, regardless of stimulus type.

The significance of stimulus type [F(2, 104) = 443, *p* < 0.00001, ε = 0.999] indicated faster reading times for words than for nonwords and for text than for words. In summary, musicians were more fluent readers than controls, regardless of ability level and stimulus type. This effect is rather surprising in view of the self-reported reading habits, which indicated that controls read twice as many books (in text) as musicians in one year. However, the number of pages read by the musicians did not take into account the number of pages of sheet music, and thus underestimated the massive attention-eye shifting exercise involved in reading sheet music, which is inherent in being a professional musician.

### Behavioural data

#### Accuracy

The ANOVA performed on the percentages of correct responses during the EEG recording showed the significance of the group factor [F (1, 52) = 27.5, *p* < 0.000003; η_p_^2^ = 0.35] with higher accuracy in musicians than in controls regardless of proficiency and target type (MUS = 98.5%, SD = 0.67; CON = 93.3%, SD = 0.7). The further significance of target type factor [F (1, 52) = 37.56, *p* < 0.000001, ε = 1; η_p_^2^ = 0.42] indicated a higher accuracy for letters (98.78%, SD = 0.1) than notes (92.98%, SD = 0.96). The significant interaction of group x target type [F (1, 52) = 28.82, *p* < 0.000002, ε = 1; η_p_^2^ = 0.36] showed a much lower accuracy for notes than for letters in controls (notes = 87.87%, SD = 1.41; letters = 98.82%, SD = 0.14; *p* < 0.0001), no difference in accuracy for the 2 target types in musicians (notes = 98.1%, SD = 1.29; letters = 98.82%, SD = 0.14), and no difference in accuracy with letters between groups (musicians = 98.82%, controls = 98.74%, SD = 0.16).

#### Reaction times (RTs)

**Figure 4:**
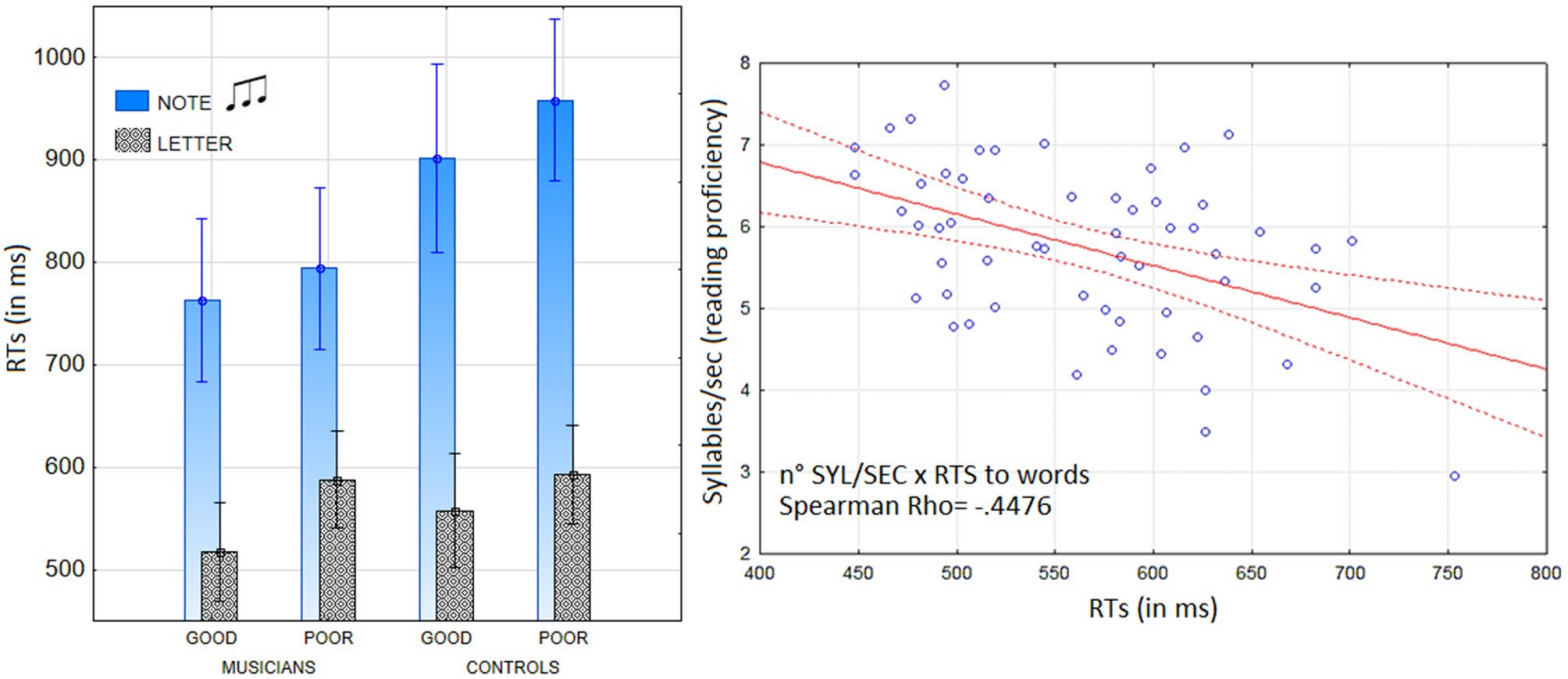
(Left) RTs analyses showed how good word readers were also good at detecting notes, regardless of musicianship. (Right) Scatterplot depicting the significantly negative correlation between the results of the three reading tests and word response times in all participants.

The ANOVA performed on the mean response times during the EEG recording showed the significance of group factor [F (1, 52) = 19.83, *p* < 0.00005; η_p_^2^ = 0.28] with faster RTs in musicians (665.5 ms, SD = 13.2) than in controls (753.8, SD = 14.4). Further significance of proficiency factor [F (1, 52) = 6.15, *p* < 0.016; η_p_^2^ = 0.11] showed faster RTs in good readers (684.8 ms, SD = 14.4) than in poor readers (733.5 ms, SD = 13.27), regardless of target type. The factor target type also yielded significance [F (1, 52) = 430, *p* < 0.00001; ε = 1; η_p_^2^ = 0.89] with faster RTs to letters (564 ms, SD = 8.79) than notes (854.4 ms, SD = 14.6). The ANOVA also showed the significant interaction of group x target type [F (1, 52) = 21.13, *p* < 0.00003; ε = 1; η_p_^2^ = 0.29]. Post-hoc comparisons showed no difference in the groups’ mean RTs to letters, although musicians tended to be faster than controls in word reading, and musicians showed a strong advantage over controls in the note detection condition (see Fig. 4, left).

Correlation analyses were carried out between the reading test scores (word reading proficiency) and word response times in the experimental task. The two variables were found to be negatively significantly correlated, (Rho = − 0.4476, *p* < 0.05 in the whole sample), as can be seen in Fig. 4 (right). The correlation was more significant for musicians (Rho = − 0.56, *p* < 0.05) than for controls (Rho = − 0.4, *p* < 0.05).

### Electrophysiological data

#### N170 component

**Figure 5:**
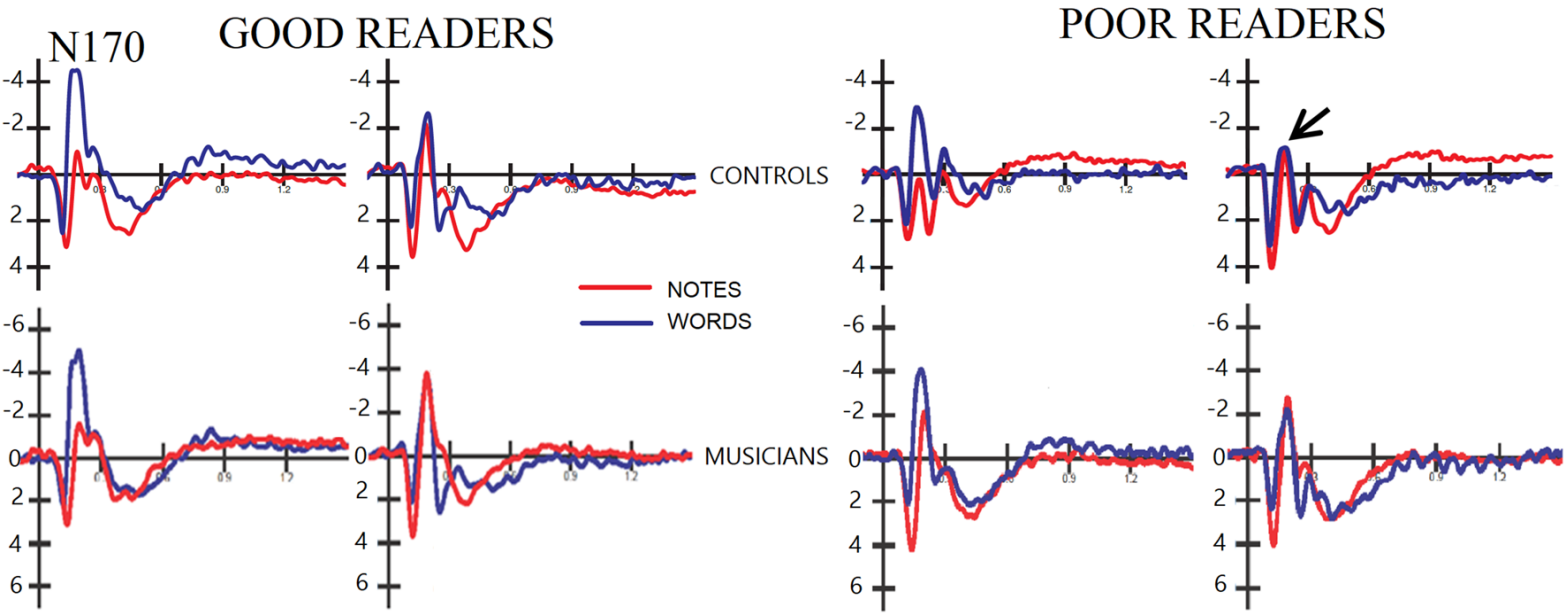
Grand-average ERP waveforms recorded at left and right occipito/temporal sites in musicians vs. controls as a function of their reading proficiency. Poor readers showed a smaller right-sided hemispheric N70 response, especially if non-musicians.

**Figure 6:**
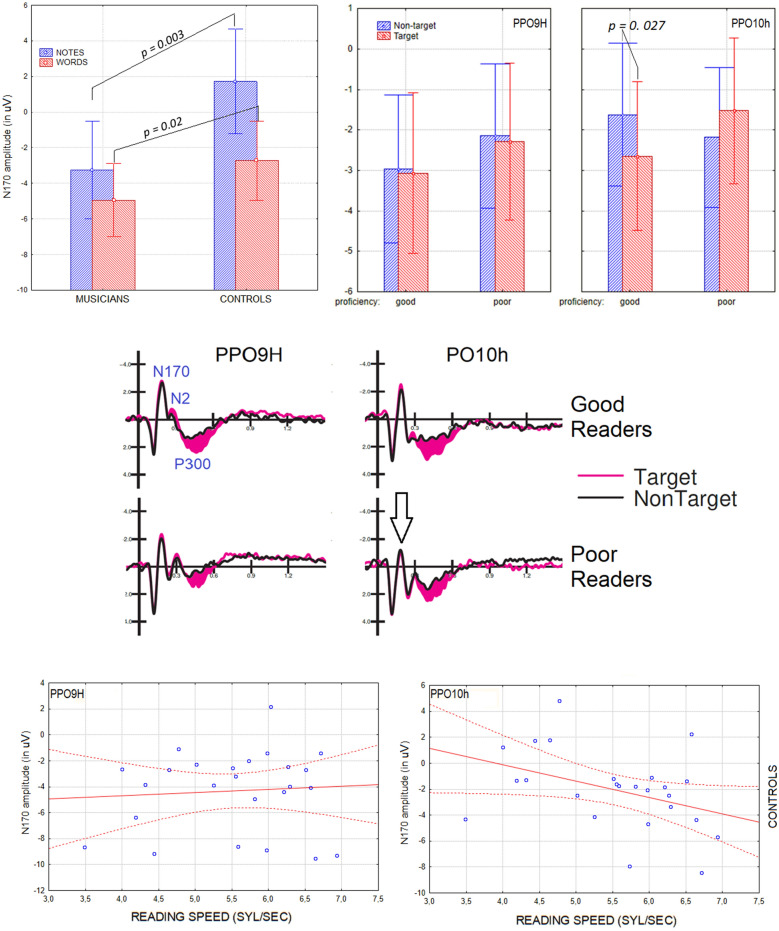
(Top) Left: Interaction between musicianship and stimulus type. Mean amplitude values of N170 response recorded in the two groups of participants as a function of stimulus type (notes vs. words). Right: Interaction between targetness x hemisphere x proficiency. Mean amplitude values of N170 response recorded in the two groups of poor and good readers over the left and right occipito/temporal areas, in response to targets and non-targets. (Middle) Grand-average ERP waveforms recorded at left and right occipito/temporal sites in controls in response to target vs. non-target stimuli as a function of their reading proficiency. (Bottom) Correlational analyses between N170 amplitudes in response to words and word reading speed (syl/sec) in controls.

The ANOVA performed on the amplitudes of the N170 component in the time window between 170 and 210 ms at PPO9h and PPO10h electrodes for good and poor readers (musicians vs. controls, respectively) showed the significance of the group factor with larger N170 responses recorded in musicians (− 4.11 µV, SE = 0.54) than in controls (− 0.50 µV, SE = 0.58), regardless of stimulus type or proficiency [F (1, 52) = 20.75, *p* < 0.00003; η_p_^2^ = 0.28]. The ANOVA also revealed the stimulus type factor [F (1, 52) = 58.1, *p* < 0.00001; ε = 1; η_p_^2^ = 0.53], with larger responses to words (− 3.83 µV, SE = 0.38 than to notes (− 0.77 µV, SE = 0.50), as shown in the ERP waveforms of Fig. 5. The interaction of group factor x stimulus type was also significant [F (1, 52) = 11.7, *p* < 0.001; ε = 1; η_p_^2^ = 0.50]. Post-hoc comparisons showed significantly larger N170 responses to notes in musicians than controls (*p* < 0.0003), and significantly larger responses to words in musicians than controls (*p* < 0.02), regardless of proficiency (see graph in Fig. 6 for means and SE values). There was a significant stimulus x hemisphere interaction [F (1, 52) = 20.5, *p* < 0.00004; ε = 1; η_p_^2^ = 0.28]. Post-hoc tests revealed a strong left-hemisphere asymmetry for the N170 for words (left = − 4.84 µV, SE = 0.46, right = − 2.83 µV, SE = 0.41) and a tendency for the N170 to be larger over right than left sites for notes (left = − 0.39 µV, SE = 0.57; right = − 1.16 µV, SE = 0.55).

**Figure 7:**
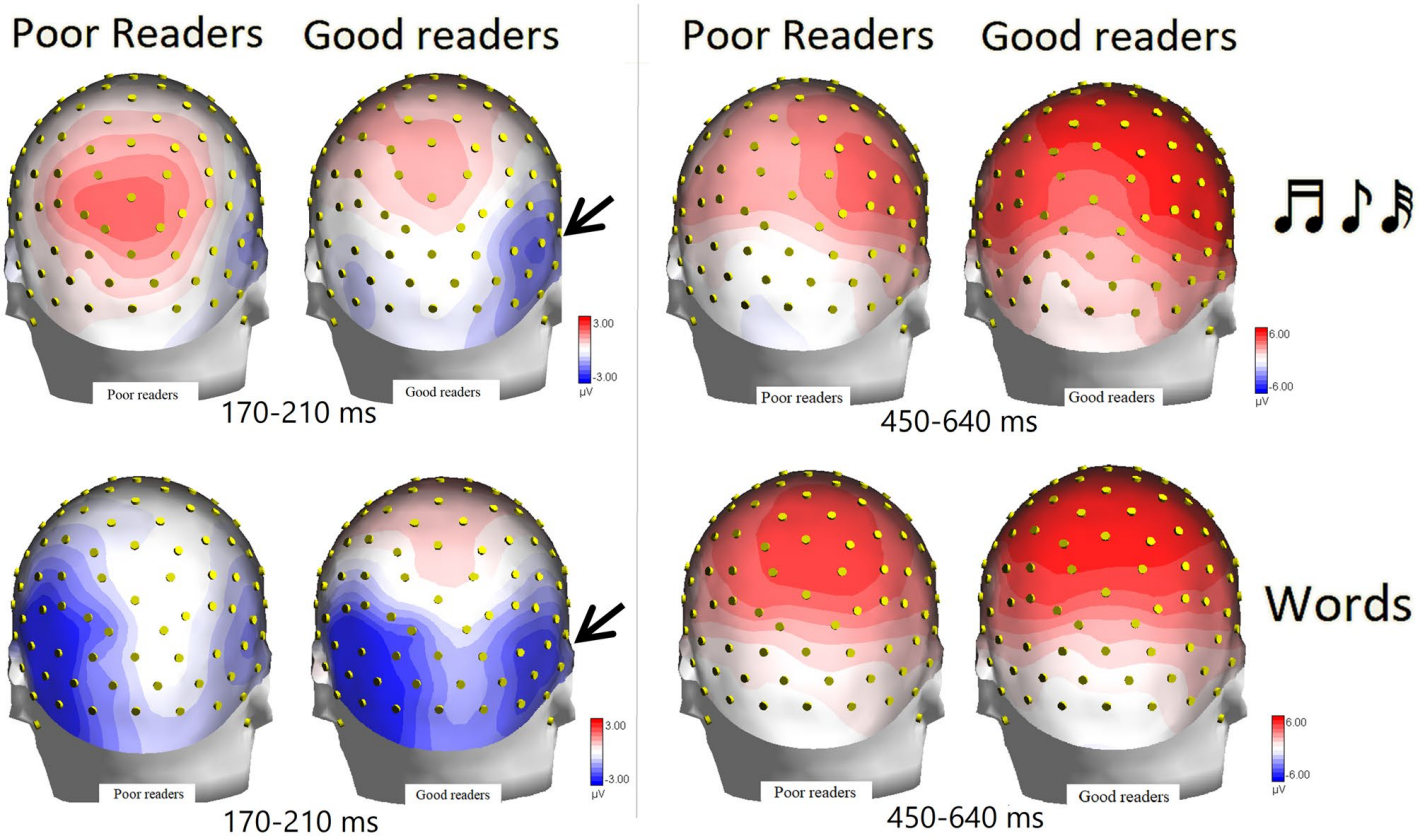
(Left) Isocolour topographical maps of N170 voltage distribution as recorded in good and poor readers (regardless of musicianship), in response to notes (upper row) and words (lower row). (Right) Same but recorded in the P300 range.

The further interaction of targetness x hemisphere x proficiency [F (1, 52) = 5.62, *p* < 0.001; ε = 1; η_p_^2^ = 0.10]. Post-hoc comparisons showed an overall larger N170 in the left than in the right hemisphere, in good than in poor readers regardless of hemisphere, and a proficiency effect in the right hemisphere, with larger responses to targets over the right hemisphere in good than in poor readers, regardless of musicianship (see graph in Fig. 6 for means and SE values). However, the further interaction of musicianship x targetness x hemisphere x proficiency [F (1, 52) = 20.5, *p* < 0.00004; ε = 1; η_p_^2^ = 0.12] showed that this effect was more true for controls (*p* < 0.001), as musicians showed a bilateral N170 response in all conditions, except for a tendency for poor readers to show a larger N170 to non-targets over the left than the right hemisphere (RH, *p* = 0.056). The topographic maps in Fig. 7a show the scalp distribution of the reading-specific N170 response, and clearly show the interaction between proficiency and hemispheric lateralization of the N170, with good readers also showing a N170 focus over the RH.

Correlation analyses were performed between individual values of N170 recorded in response to words in the left and right hemispheres and proficiency as measured by syllable/sec reading speed. In controls, there was no correlation (Rho = 0.07) between the two measures for the left hemisphere and an inverse correlation for the right hemisphere (the more negative the N170 response, the higher the proficiency; Rho = − 0.40, *p* < 0.05).

#### P300 component

**Figure 8:**
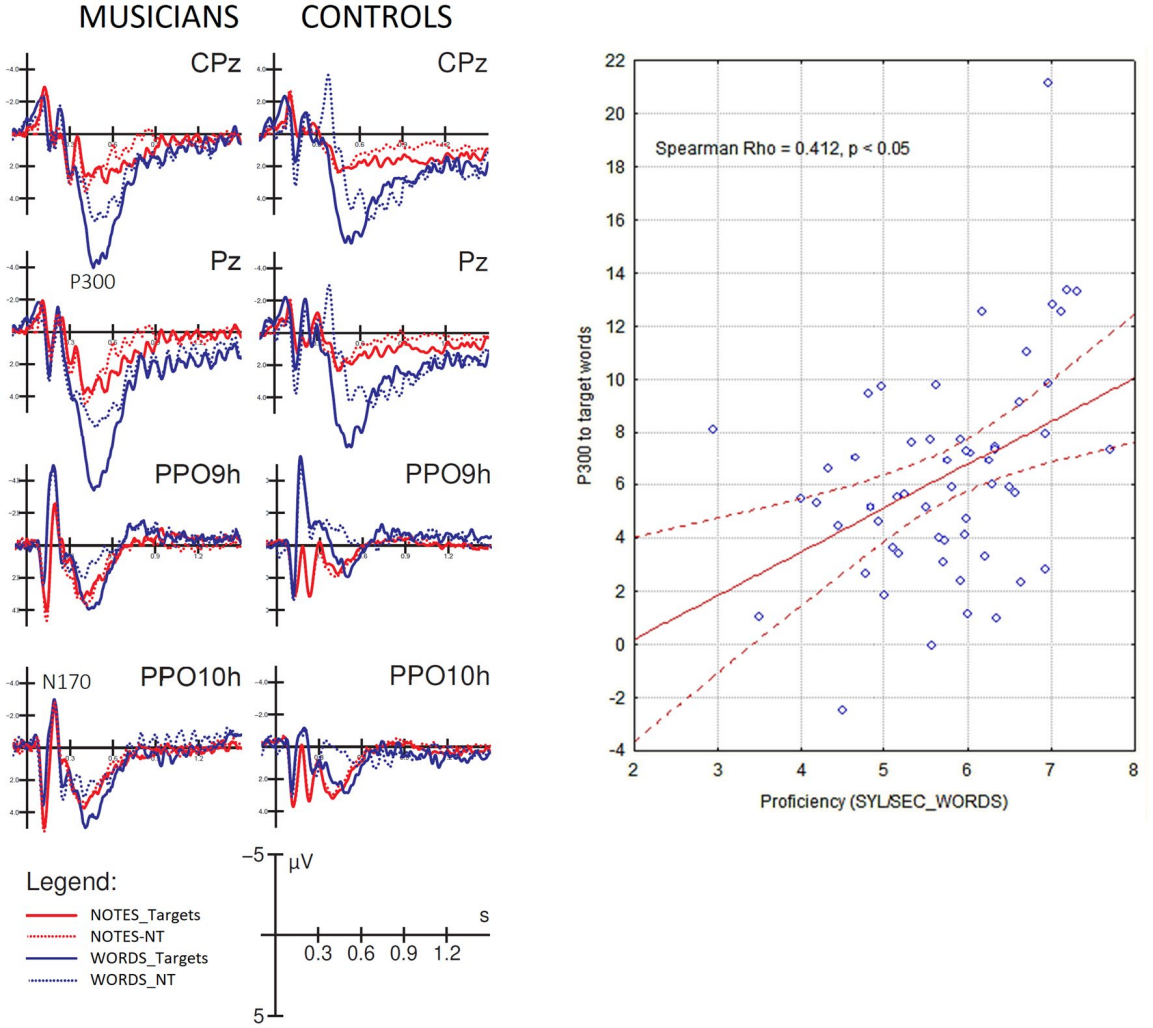
(Left) Grand-average ERP waveforms recorded at midline central, parietal and left and right occipito/temporal sites, in musicians and controls in response to notes and words when target or non-target. (Right). Correlation between P300 amplitude values in response to target words and reading proficiency for the whole sample of participants.

The ANOVA performed on the amplitudes of the centro/parietal P300 component recorded in the 450–650 ms time window at CP1 and CP2 sites showed the significance of stimulus factor [F (1, 52) = 26.7, *p* < 0.00001; ε = 1; η_p_^2^ = 0.34], with larger P300 amplitudes to words (4.90 µV, SE = 0.45) than notes (2.39 µV, SE = 0.38) in all participants, regardless of proficiency or musicianship (Fig. 7, right). Also significant was the group x proficiency interaction [F (1, 52) = 4.61, *p* < 0.0036; ε = 1; η_p_^2^ = 0.08]. Post-hoc comparisons showed larger P300 recorded in good (5.0 µV, SE = 0.62) than poor musician readers (3.0 µV, SE = 0.66, *p* < 0.03), but no difference between the P300 recorded in good (2.84 µV, SE = 0.69) than in poor (3.74 µV, SE = 0.69) control readers. Post-hoc comparisons also showed larger P300 responses recorded in good musicians vs. good control readers (*p* < 0.02), but no difference between P300 responses recorded in poor musicians vs. poor controls. The ANOVA also revealed the significance of the stimulus x group interaction [F (1, 52) = 8.1, *p* < 0.006; ε = 1; η_p_^2^ = 0.14]. Post-hoc comparisons showed larger P300 to words in musicians (5.95 µV, SE = 0.51) than in controls (2.72 µV, SE = 0.55, *p* < 0.01), as can be seen from the ERP waveforms in Fig. 8 (left). The stimulus targetness factor was also found to be significant [F (1, 52) = 36.5, *p* < 0.00001; ε = 1; η_p_^2^ = 0.41], with larger P300s to target (4.46 µV, SE = 0.38) than non-target stimuli (2.84 µV, SE = 0.34). The significant interaction of targetness x group [F (1, 52) = 4.27, *p* < 0.04; ε = 1; η_p_^2^ = 0.08] indicated the P300 was larger to targets than to non-targets in both groups, with a tendency for the P300 to be larger to targets in musicians than in controls (*p* = 0.06). Finally the ANOVA revealed the significance of stimulus type x targetness [F (1, 52) = 17.1, *p* < 0.0001; ε = 1; η_p_^2^ = 0.25]. Post-hoc comparisons showed larger P300s to target than non-target stimuli, regardless of stimulus type, but with a larger effect for words (target = 6.21 µV, SE = 0.49; non-target = 3.61 µV, SE = 0.48; *p* < 0.0001) than for notes (target = 2.71 µV, SE = 0.43; non-target = 2.07 µV, SE = 0.39; *p* < 0.04). Correlation analyses between individual amplitude values of the P300 component in response to target words and word reading speed (syllables per second) showed the significance of the relationship in both musicians and controls (r = 0.412, *p* < 0.05), as can be seen in Fig. 8 (right).

### Source reconstruction

#### N170 swLORETA

**Figure 9:**
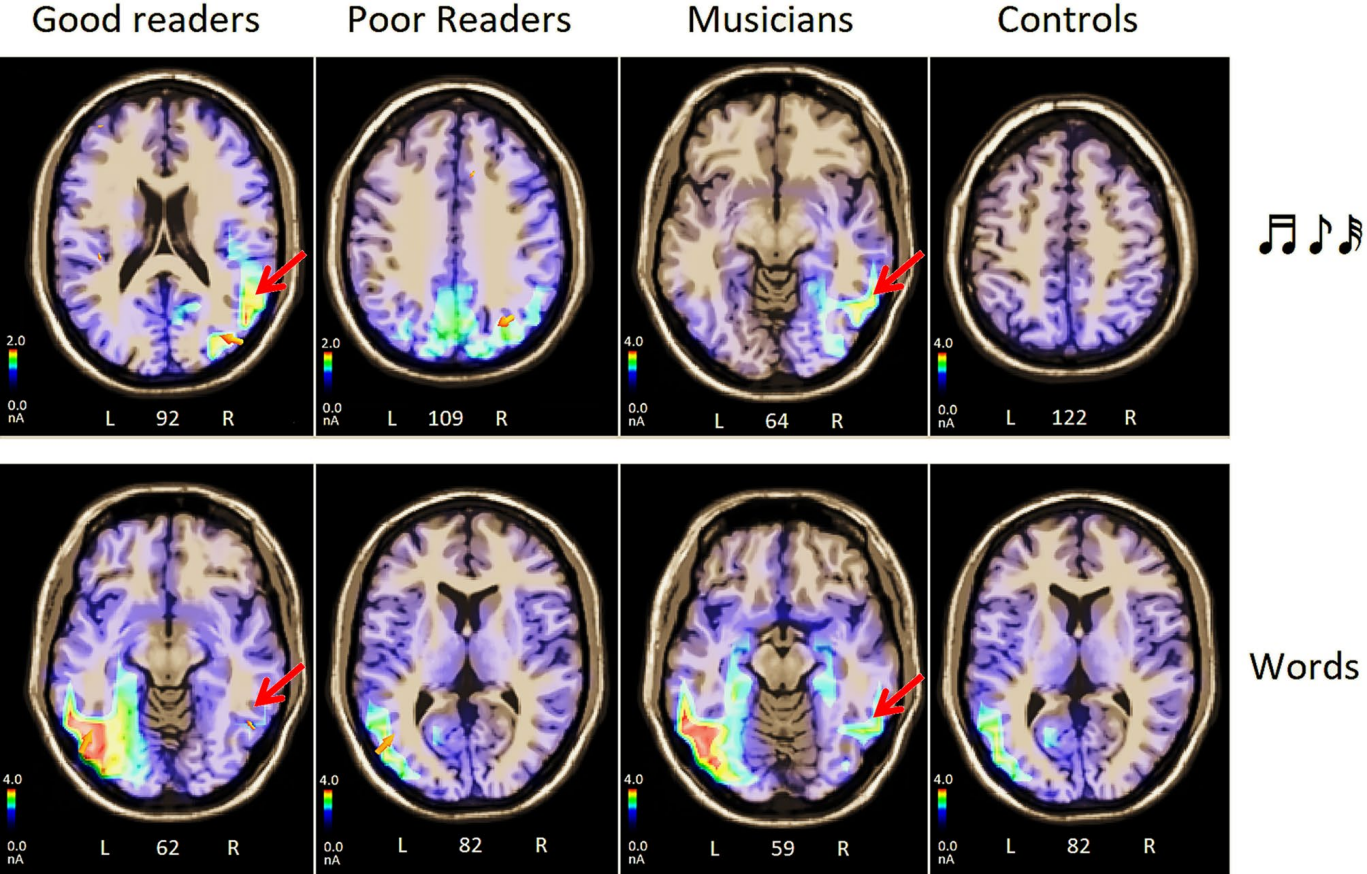
Axial brain sections showing the location and strength of electromagnetic dipoles explaining the surface voltage of N170 response (170–210 ms), in musicians vs. controls, in good vs. poor readers, as a function of stimulus type (notes vs. words).

**Figure 10:**
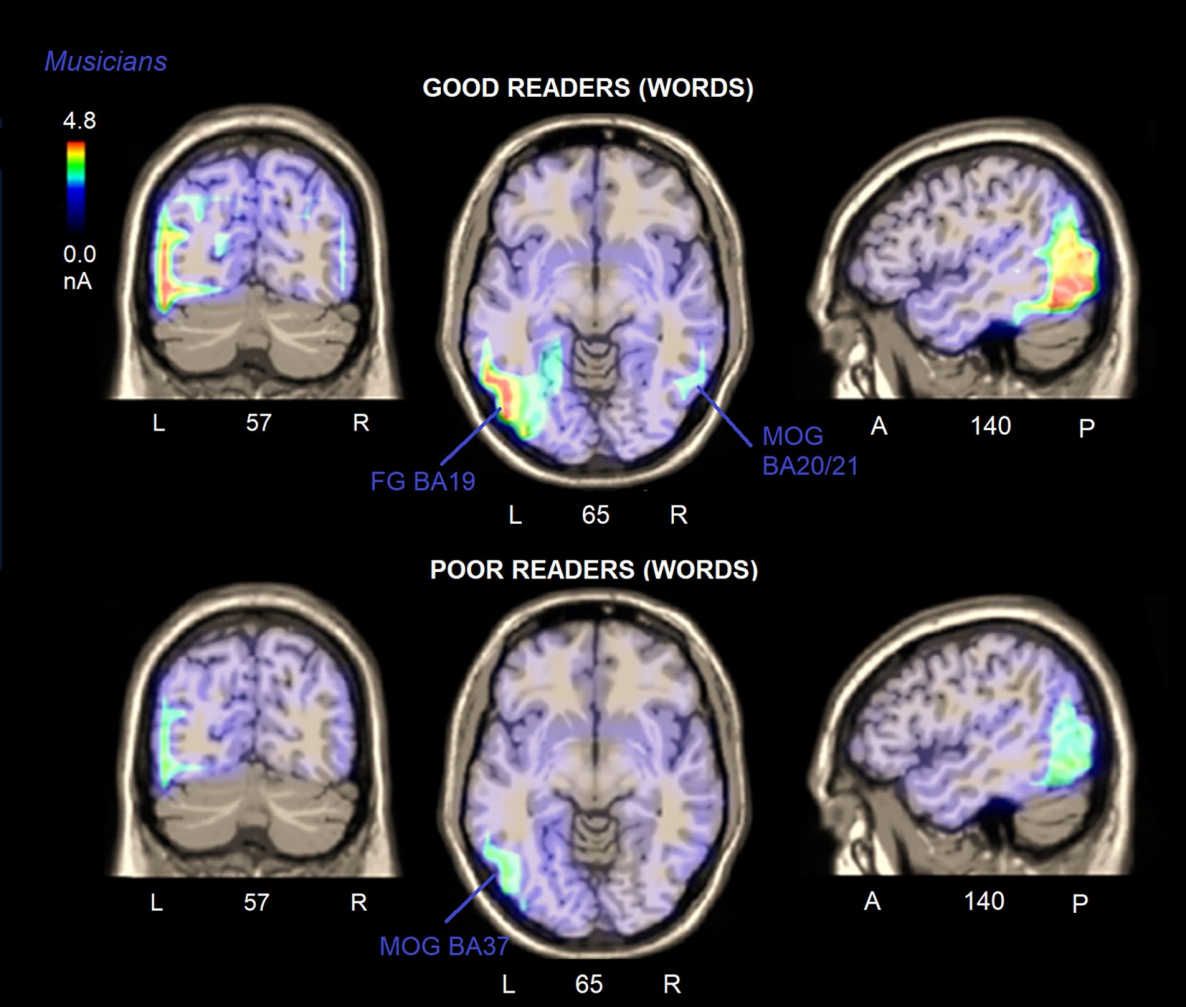
Coronal, axial and sagittal brain sections showing the location and strength of electromagnetic dipoles explaining the surface voltage of N170 response (170–210 ms) to words, in musicians as a function of their reading proficiency (good vs. poor readers). A higher reading proficiency was associated with a right-hemispheric involvement of OT cortex in reading.

Eight swLORETA (*standardised weighted low resolution electromagnetic tomography*) source reconstructions were performed on the scalp-recorded voltage of N170 component (170–210 ms) as recorded in groups of poor or good readers, musicians or controls, in response to notes or words. The inverse solutions are shown in Figs. 9 and 10, while Table 2 lists the corresponding active electromagnetic dipoles according to the specific comparison.

**Table 2:** N170—List of active electromagnetic dipoles (along with their Talairach coordinates and relative Brodmann areas) explaining the scalp‐recorded potentials measured in the 170–210 ms time window in response to notes and words, in good and poor readers (regardless of musicianship) or in musicians and controls (regardless of proficiency).

Magn	x [mm]	Y [mm]	Z [mm]	H	Gyrus	BA
Notes (good readers)						
12.05	40.5	− 78.5	19.5	**R**	**Middle Occipital Gyrus**	**19**
8.38	40.5	11.5	29.5	R	Precentral Gyrus	6
7.66	0.5	− 18.5	29.5	R	Cingulate Gyrus	23
6.89	10.5	11.5	69.5	R	Superior Frontal Gyrus	6
6.86	− 29.5	− 18.5	− 40.5	L	Uncus	20
6.77	10.5	51.5	49.5	R	Superior Frontal Gyrus	8
6.74	− 39.5	− 28.5	19.5	L	Superior Temporal Gyrus	41
6.69	20.5	61.5	29.5	R	Superior Frontal Gyrus	9
6.11	− 9.5	61.5	− 20.5	L	Superior Frontal Gyrus	10
5.80	− 49.5	21.5	− 20.5	L	Superior Temporal Gyrus	38
5.40	− 39.5	51.5	19.5	L	Middle Frontal Gyrus	10
5.27	0.5	41.5	− 30.5	R	Medial Frontal Gyrus	11
5.14	− 49.5	31.5	29.5	L	Middle Frontal Gyrus	9
Notes (poor readers)						
11.60	30.5	− 68.5	29.5	**R**	**Precuneus**	**31**
5.58	10.5	21.5	29.5	R	Cingulate Gyrus	24
5.07	0.5	11.5	69.5	R	Superior Frontal Gyrus	6
Words (good readers)						
14.68	− 49.5	− 68.5	− 10.5	**L**	**Fusiform Gyrus**	**19**
12.29	50.5	− 58.5	9.5	**R**	**Middle Temporal Gyrus**	**21**
12.26	50.5	− 58.5	− 10.5	**R**	**Inferior Temporal Gyrus**	**20**
12.02	20.5	− 28.5	− 20.5	R	Parahippocampal Gyrus	35
11.43	− 39.5	51.5	19.5	L	Middle Frontal Gyrus	10
11.40	0.5	− 18.5	29.5	R	Cingulate Gyrus	23
11.36	− 9.5	61.5	− 20.5	L	Superior Frontal Gyrus	10
11.35	− 9.5	41.5	− 30.5	L	Rectal Gyrus	11
11.10	0.5	11.5	69.5	R	Superior Frontal Gyrus	6
9.76	10.5	51.5	49.5	R	Superior Frontal Gyrus	8
9.40	− 39.5	31.5	39.5	L	Precentral Gyrus	9
9.40	− 19.5	31.5	59.5	L	Superior Frontal Gyrus	6
8.86	0.5	61.5	29.5	R	Medial Frontal Gyrus	9
7.96	40.5	11.5	29.5	R	Precentral Gyrus	6
3.44	− 49.5	− 68.5	9.5	L	Middle Occipital Gyrus	37
1.30	50.5	− 68.5	19.5	R	Middle Temporal Gyrus	39
1.24	20.5	− 8.5	− 30.5	R	Parahippocampal Gyrus	35
Words (poor readers)						
13.44	− 49.5	− 68.5	9.5	**L**	**Middle Occipital Gyrus**	**37**
11.30	50.5	− 68.5	19.5	R	Middle Temporal Gyrus	39
11.24	20.5	− 8.5	− 30.5	R	Parahippocampal Gyrus	35
11.06	− 9.5	61.5	− 20.5	L	Superior Frontal Gyrus	10
11.06	0.5	− 8.5	29.5	R	Cingulate Gyrus	23
7.86	− 19.5	41.5	49.5	L	Superior Frontal Gyrus	8
7.73	− 39.5	51.5	19.5	L	Middle Frontal Gyrus	10
Notes (musicians)						
**13.57**	50.8	− 68	4.7	**R**	**Middle Occipital Gyrus**	**37**
**11.82**	− 48.5	− 78.2	3.8	**L**	**Middle Occipital Gyrus**	**19**
11.28	− 28.5	− 15.3	− 29.6	L	Uncus	20
11.06	11.3	40.5	50.7	R	Superior Frontal Gyrus	8
11.06	11.3	52.4	33.7	R	Superior Frontal Gyrus	9
11.00	40.9	2.4	29.4	R	Precentral Gyrus	6
9.72	− 38.5	− 28.5	17.1	L	Superior Temporal Gyrus	41
8.97	− 8.5	57.3	− 9	L	Superior Frontal Gyrus	10
8.21	− 8.5	64.4	16.8	L	Superior Frontal Gyrus	10
8.02	1.5	− 1.1	65	R	Superior Frontal Gyrus	6
7.71	1.5	38.2	− 17.9	R	Medial Frontal Gyrus	11
7.06	− 38.5	− 21	35.7	L	Postcentral Gyrus	3
5.09	− 38.5	21.4	40	L	Precentral Gyrus	9
Notes (controls)						
**11.42**	11.3	− 72	40.3	**R**	**Precuneus**	**7**
7.22	− 48.5	− 47.8	6.4	L	Superior Temporal Gyrus	22
6.17	1.5	− 1.1	65	R	Superior Frontal Gyrus	6
5.10	− 58.5	3.3	20.5	L	Precentral Gyrus	6
5.82	11.3	33.4	23.1	R	Anterior Cingulate	32
4.51	1.5	38.2	− 17.9	R	Superior Frontal Gyrus	6
4.94	− 8.5	57.3	− 9	L	Superior Frontal Gyrus	10
Words (musicians)						
**15.25**	− 48.5	− 66.1	− 10.9	**L**	**Fusiform Gyrus**	**19**
**13.28**	50.8	− 57.9	5.6	**R**	**Middle Temporal Gyrus**	**21**
**13.28**	50.8	− 55.9	− 10.2	**R**	**Inferior Temporal Gyrus**	**20**
12.77	31	− 24.5	− 15.5	R	Parahippocampal Gyrus	35
11.83	− 8.5	57.3	− 9	L	Superior Frontal Gyrus	10
11.73	1.5	38.2	− 17.9	R	Medial Frontal Gyrus	11
11.72	1.5	52.4	33.7	R	Medial Frontal Gyrus	9
11.70	1.5	40.5	50.7	R	Superior Frontal Gyrus	8
11.63	1.5	− 20.3	26.8	R	Cingulate Gyrus	23
11.36	− 38.5	43.4	23.9	L	Middle Frontal Gyrus	10
11.04	1.5	− 1.1	65	R	Superior Frontal Gyrus	6
9.97	40.9	2.4	29.4	R	Precentral Gyrus	6
8.95	31	− 7	46.3	R	Middle Frontal Gyrus	6
7.50	− 38,5	21.4	40	L	Precentral Gyrus	9
Words (controls)						
**13.46**	− 48.5	− 68	4.7	**L**	**Middle Occipital Gyrus**	**37**
11.5	1.5	− 20.3	26.8	R	Cingulate Gyrus	23
11.04	40.9	− 12.3	18.8	R	Insula	13
11.01	21.2	− 16.8	− 14.8	R	Parahippocampal Gyrus	28
9.02	40.09	2.4	29.4	R	Precentral Gyrus	6
9.0	31	− 7	46.3	R	Middle Frontal Gyrus	6
8.38	50.8	− 33.7	− 23.6	R	Fusiform Gyrus	20
8.25	21.2	52.4	33.7	R	Superior Frontal Gyrus	9
8.17	− 8.5	38.2	− 17.9	L	Rectal Gyrus	11
7.85	− 8.5	57.3	− 9	L	Superior Frontal Gyrus	10
7.12	− 38.5	43.4	23.9	L	Middle Frontal Gyrus	10

The strongest sources of activity for the various conditions are in bold.

The strength of electromagnetic dipoles (magnitude) is expressed in nA (nanoamperes).

Magn, magnitude; H, hemisphere; BA, Brodmann areas.

#### Good vs. poor readers

Electromagnetic dipoles significantly active during word processing for good readers were the left fusiform gyrus (BA19) and the right middle (BA21) and inferior (BA20) temporal gyri (See Fig. 10). In contrast, the most active dipoles in poor readers were the left middle occipital gyrus (MOG, BA37), and no other right-sided visual regions.

Compared to good readers, poor readers had fewer active regions, not including the dorsolateral (BA9) and superior, left and right frontal (BA6) prefrontal cortex. Poor readers also showed significantly lower activations than good readers in areas involved in orthographic encoding (left BA37/19), reading (BA35), executive systems and attention (BA10 superior, BA10 middle), response selection (BA23), and eye movements (frontal eye fields FEF, BA8), as shown by the significance of non-parametric tests (Sign test: z = 2.04, *p* = 0.04, Wilcoxon test: z = 2.2, *p* = 0.028).

Electromagnetic dipoles that were significantly active during note processing for good readers were the right MOG (BA19) and for poor readers the right precuneus. Compared to good readers, poor readers had significantly fewer active regions, not including regions involved in shifting attention and eye movements (SFG, BA8), auditory processing in sensory (BA41) and associative (BA38) areas, executive systems, working memory and attention (bilateral BA 6, 9, 10, 11). Poor readers also showed significantly less activation than good readers in the cingulate gyrus (BA24) and SFG (BA6).

#### Musicians vs. controls

The electromagnetic dipoles that were significantly active during word processing for musicians were the left fusiform gyrus (FG BA19) and the right middle (BA21) and inferior (BA20) temporal gyrus (Fig. 10). In contrast, the most active visual dipoles in controls were the left middle occipital gyrus (MOG, BA37), and the right FG BA20. Compared to musicians, controls showed significantly weaker activations in areas involved in orthographic analysis (left FG BA19/37 and right FG BA20) reading (BA35 and BA38), response selection (BA23), executive systems, working memory and attention (i.e., left SFG BA10), right MFG (BA6), right dorsolateral prefrontal cortex (BA9, left superior and middle frontal gyri (BA10/11)), as demonstrated by the statistical significance of non-parametric tests applied to the observed magnitudes (Sign test: z = 2.0, *p* = 0.045, Wilcoxon test: z = 2.55, *p* = 0.01).

Electromagnetic dipoles that were significantly active during note processing in musicians were the right MOG BA37 and the left MOG BA19, while in controls it was the right precuneus BA7 (Fig. 9). Compared to musicians, controls had significantly fewer active regions, not including regions involved in auditory processing (Heschl gyri BA41), shifting of attention and eye movements (FEF BA8), executive systems, working memory and attention (right superior and middle frontal gyri, BA9 and BA11). Compared to musicians, controls showed significantly weaker activations in the superior temporal gyrus (BA22) and in the left and right superior frontal gyrus (BA6 and BA10), as shown by the significance of non-parametric tests (Sign test: z = 1.8, *p* = 0.07, Wilcoxon test: z = 2.02, *p* = 0.04).

#### P300 swLORETA

Two swLORETA source reconstructions were performed on the scalp-recorded voltage of P300 component (450–650 ms) as recorded in groups of musicians and non-musicians during word processing. Table 3 lists the corresponding active electromagnetic dipoles found in the two groups of readers.

**Table 3:** P300—List of active electromagnetic dipoles (along with their Talairach coordinates and relative Brodmann areas) explaining the scalp‐recorded potentials measured in the 450–650 ms time window in response words, in musician and control readers.

Magn	x [mm]	Y [mm]	Z [mm]	H	Gyrus	BA	Function
Musicians							
5.34	− 59.5	− 38.5	39.5	L	Supramarginal	40	Visual word recognition
3.7	− 29.5	− 88.5	19.5	L	Middle Occipital	18	Orthographic processing
3.61	− 29.5	31.5	49.5	L	Superior Frontal	8	Ocular/attentive shifting
3.21	60.5	1.5	− 10.5	R	Middle Temporal	21	Orthographic processing
2.81	10.5	51.5	49.5	R	Superior Frontal	8	Ocular/attentive shifting
2.79	0.5	− 48.5	19.5	R	Posterior Cingulate	30	Response selection
2.72	0.5	− 28.5	29.5	R	Cingulate Gyrus	23	
2.5	− 69.5	− 38.5	− 0.5	L	Middle Temporal	21	Orthographic processing
2.45	− 59.5	− 58.5	− 0.5	L	Inferior Temporal	37	Orthographic processing
2.36	− 9.5	1.5	− 40.5	L	Uncus		
1.6	40.5	− 88.5	− 10.5	R	Inferior Occipital	18	Orthographic processing
Controls							
3.43	− 59.5	− 38.5	39.5	L	Supramarginal Gyrus	40	Visual word recognition
2.44	− 29.5	− 88.5	29.5	L	Middle Occipital	19	Orthographic processing
2.4	40.5	21.5	− 30.5	R	Superior Temporal	38	
2.39	0.5	− 78.5	39.5	R	Cuneus	19	Orthographic processing
2.3	− 69.5	− 38.5	− 0.5	L	Middle Temporal	21	Orthographic processing
2.21	10.5	41.5	49.5	R	Superior Frontal	6	
2.0	− 9.5	1.5	− 40.5	L	Uncus	28	
1.79	− 29.5	31.5	49.5	L	Superior Frontal	8	

The strength of electromagnetic dipoles (magnitude) is expressed in nA (nanoamperes).

Magn, magnitude; H, hemisphere; BA, Brodmann areas.

Compared to musicians, controls had significantly fewer active regions, not including right hemispheric orthographic areas (BA18 and BA21) and regions involved in target selection and non-target suppression (the cingulate and posterior cingulate cortices BA 30 and BA23), as well as in shifting of attention and eye movements (FEF, BA8). Compared to controls, musicians showed significantly stronger cerebral activations in the left supramarginal gyrus (BA40) devoted to visual word recognition and in the left VWFA.

## Discussion

The main aim of the present study was to investigate the neural correlates of note and word reading in people with different music proficiency to investigate how musical skills can influence and modify word reading ability and mechanisms. To this end, a sample of graduate non-musicians and musicians with a degree or diploma from a conservatoire was recruited. A word, non-word and text reading task was first administered to the participants. Event-related potentials were recorded during a letter/note detection task. Preliminary reading tests, showed how musicians were more proficient in language reading than controls. They were able to pronounce a higher number of syllable/sec than controls in all reading tests (word, non-word and text), regardless of the instrument played. Reading tests showed to be highly correlating among each other and with response times to target words recorded in the experimental session, which supports their reliability, and that of experimental paradigm in assessing reading proficiency. The higher reading proficiency of musicians, expected on the basis of previous literature (e.g.,^24–31,34^), was somewhat surprising given the self-reported reading habits, according to which musicians were used to read less than half as many books per year as non-music students. However, these estimates did not take into account the large number of musical scores that professional musicians read over the course of a year, which, if not full of words, are rich in symbols and equally stimulate reading, attention and ocular mechanisms^44,66^. Based on reading tests, the sample was further subdivided into poor and good readers, who coherently showed slower RTs to words and notes in the former and faster RTs to words and notes in the latter. The fact that word ability predicted the ability to detect notes embedded in musical bars suggests the existence of a common reading mechanism that benefits from musical literacy^34^.

ERP data showed larger orthographic N170 responses to words than to notes, larger N170 responses in good readers than in poor readers, and in musicians than in controls. The N170 component was found to be larger for word than for note recognition. These results are consistent with empirical evidence confirming the role of the occipitotemporal N170 in reflecting word recognition processes^67,68^. An interesting finding from the analysis of the N170 component is the significant interaction of the stimulus factor with the hemisphere factor. From the results, it would appear that there was not much difference in hemispheric activation between notes and words in the right hemisphere. On the contrary, the literature on the orthographic N170 provides evidence demonstrating that the processing of the orthographic properties of linguistic stimuli takes place in the VWFA, located in the left inferotemporal cortex^69–72^.

In this study, analyses showed a strong left-hemispheric asymmetry for N170 during word processing and a tendency for N170 to being larger over right than left sites to notation. The further interaction of targetness x hemisphere x proficiency showed overall larger N170s in the left than in the right hemisphere, in good than poor readers regardless of hemisphere, and a proficiency effect on the right hemisphere, with larger response to targets over the right hemisphere in good than poor readers, regardless of musicianship. This suggests the key role of right hemispheric specialization in enhanced reading skills. Again, the interaction of musicianship x targetness x hemisphere x proficiency showed that this effect was more pronounced for controls since musicians already showed a bilateral N170 response in all conditions, which correlated with their increased reading skills.

Correlation analyses performed between individual values of N170 to words and syllable/sec reading speed showed no correlation in controls over the left hemisphere, but a significant inverse correlation over the right hemisphere (the more negative the N170 response, the higher the reading proficiency). This finding further supports the causal hypothesis that an intensive training and specialization of the right orthographic area might lead to a superior performance (in good vs. poor readers, and in musicians vs. controls). This finding may suggest that early musical literacy in reading music does indeed lead to right hemisphere involvement in visual word processing. Right-hemisphere involvement in reading music notation is a robust notion widely supported by the previous literature^34,38–45^. The evidence for a difference in activation of the right fusiform gyrus in response to words between good and poor readers highlights the neuroplastic effects of music education on visual word recognition ability. The swLORETAs applied to N170 component, which were carried out on groups of musician and control readers, showed, among other things, an effect of musical literacy and proficiency on the lateralisation of the reading neural mechanism.

In addition, the results provide evidence for a reduced ability to discriminate stimuli in poor readers, as evidenced by a reduced amplitude of the P300 component, as well as slower RTs and reading times. This may be due to difficulties in sustaining attention, a poorer ability to shift attention and focus rapidly in search of the target, and a poorer ability to detect targets visually. A positive correlation was also found between P300 amplitude and reading speed (number of syllables per second). From these data, it can be concluded that shorter reading times are associated with a greater P300 amplitude, which reflects discrimination certainty^73^, due to a greater ability to discriminate and classify the stimulus, as well as attentiveness^74–76^ in musicians, as well as in good than poor readers. Indeed, overall, P300 response to words was found of greater amplitude in musician than control readers. Although the psychophysiological literature is lacking previous evidence of enhanced P300 to written words in musician as compared to non-musicians (see the previously mentioned ERP studies^34,35,44^) this effect might suggest enhanced perceptual and selective attention abilities, linked to the neuroplastic reading-related changes, such as the development of a right orthographic area (shown by N170 and behavioural data). Additionally, the constant eye movements involved in reading music might strengthen attentional shifting and reading skills. Both hypotheses were supported by the results of the source reconstruction applied to P300 potentials. The data showed stronger brain activity in musicians compared to control readers in regions involved in visual word recognition (i.e., the left supramarginal gyrus^77^), orthographic processing (the left B37 and bilateral BA18 and BA 21 areas), target selection (the posterior cingulate cortex^78^), and ocular/attentional shifting (FEF, BA8^79^). This pattern of results further suggests that musical literacy improves visual word recognition by enhancing orthographic and letter recognition areas over posterior brain areas, and by providing more efficient attentional selection mechanisms. Specularly, the reduced amplitude of the P300 in poor readers could be due to a greater difficulty in detecting the targets, due to the inefficiency of the orthographic analysis mechanism (insufficient VWFA activity and lack of bilateral activation), or due to their difficulty in maintaining sustained attention. There is some empirical evidence in the literature for the presence of a reduced amplitude of the P300 component in individuals with attentional disorders^80,81^. For example, Papagiannopoulou and Lagopoulos^82^ found a reduced amplitude of the P300 component in children with dyslexia and impaired attentional resource allocation. In light of this evidence, the data from the present study regarding the lower P300 amplitude in subjects with lower reading ability appears to be consistent with the longer response times they showed in the note and word recognition tasks.

### Word processing

In detail, during word processing, larger N170 magnitudes from VWFA sources (left fusiform/MOG BA19 and BA37) were found in good vs. poor readers (reading ability) and in musicians than in controls (musicianship and derived enhanced skills). On the other hand, a reduced activation of the VWFA was found in both poor readers and controls, suggesting less efficient orthographic processing. This is consistent with the poorest text reading performance of poor readers and their less efficient ability to detect target letters (as well as target notes). The reduced activation of the left FG in poor readers is strongly consistent with its role in reading performance.

There is literature to suggest that individuals with dyslexia have reduced VWFA activation^83,84^. At this regard, Maisog et al.^85^ conducted two meta-analyses to explore the neurological foundation of developmental dyslexia. The study found that dyslexic individuals have hypoactivity in the left extrastriate cortex, which is consistent with the results of the present investigation. The hypoactivation of the visual word form area (VWFA) and the homologous area in the right hemisphere among individuals with poor reading skills was concomitant with a diminished and less extensive neurometabolic activation in the prefrontal region. The reduced activation of the latter possibly arises from a resulting decrease in attentional capacity and working memory^86^, which may adversely affect the performance of weaker readers compared to stronger ones. There is much evidence to suggest that injury to the left occipitotemporal area can lead to impaired reading ability, including pure alexia^87,88^ or transient alexia^89^. Consistently, developmental dyslexia (in the absence of brain damage) is instead associated with insufficient or atypical activation of the left occipito-temporal ventral cortex activation for words^88,90–96^. This atypical or insufficient activity would result in smaller amplitudes of the left occipital/temporal N170 in response to words that symbols^91,97^, as compared to controls.

Most notably, the most powerful N170 source (after the VWFA) in good readers and in musicians was the right OT (middle and inferior temporal gyrus, BA20 and 21), that was not find active in poor readers and in controls. It is possible to relate the existence of the right orthographic area active during letter search to the development of a right-sided VNFA in musicians, and to the acquired enhanced reading skills.

Statistical analyses carried out on source reconstruction data showed that brain activations were more extended and more intense in good than poor readers, and in musicians than in controls. The areas of higher activity during word reading in good readers were: the parahippocampal gyrus (BA35), strongly connected with the VWFA^98,99^ and involved in reading^100^ and visuo-spatial processing^101^. Also more active were: the right middle temporal/angular gyrus (BA39) involved in grapheme-to-phoneme conversion and phonological processing^102,103^; the right cingulate cortex (BA23) engaged in target selection^104^; bilateral anterior brain areas, including the premotor cortex (BA6), dorsolateral prefrontal (BA9), middle and superior frontal cortices and frontal eye fields (BA8), know to control attentional selection, sustained attention, and attentional and ocular shifting^105–109^.

### Note processing

During note processing, skilled readers and musicians showed the highest activation in the right middle occipital gyrus (BA19), implying this region as a putative VNFA. The literature has consolidated evidence that music reading particularly engages a specialized area of the right hemisphere of the occipito/temporal region. This area has been identified in different studies as the right transverse occipital sulcus, right occipital gyrus, right inferior occipital gyrus, right occipito/temporal junction, and right fusiform gyrus. The supporting studies notably include works by Sergent et al.^38^, Schön et al.^39^, Mongelli et al.^40^, Meinster et al.^41^, Proverbio et al.^34,42^, Wond and Gauthier^43^, Li et al.^44^, and Nakada et al.^45^. However, the same mechanism was not observed in controls, because of a lack of specialization and literacy. Musical notation reading in non-musicians (and in poor-readers) was subserved by an occipito/parietal area (the right cuneus, BA19), an area devoted to the processing of spatial properties of objects. It should be noted that in novice readers, note identification relies heavily on spatial analysis of note positions on the five pentagram lines.

A significant disparity between musicians and controls was observed in the neural network activated during the processing of musical notation. The network was substantially more extensive in musicians than in controls, encompassing the right premotor cortex, as well as the right and left dorsolateral prefrontal and superior frontal cortices, which may be associated with attentional maintenance, working memory, attentional focus, and attentional shifts.

It is worth noting that significant activation of frontal eye fields (BA8), which are involved in ocular movement and shifting of attention, was found only in musicians (and in good readers). Similarly, an activation of Heschl gyri (primary auditory area, BA41) was associated with note reading only in musicians. This area is thought to subserve the representation of sounds in music reading in musicians^110^.

## Conclusions and future perspectives

Overall, the data demonstrated how musicians' improved reading proficiency discovered in independent reading tests was associated with better performance in orthographic and notation detection tasks, specifically with increased N170 and P300 amplitudes, and larger differences between targets and non-targets. From a neural standpoint, this was manifested in more extensive, bilateral activation^111^ of regions dedicated to orthographic processing, phonological processing, attentional shifting, target selection and eye movements. On the contrary, the diminished reading scores of both control and low-performing readers are linked to a unilateral and less prominent response in the N170 latency period. This is particularly associated with a decrease in activity in the VWFA, FEF, and frontal regions.

The evidence suggests that skilled readers, including musicians and good readers, show a unique connection between reading words and the right occipitotemporal cortex. It was discovered that reading proficiency was linked to the amplitude of N170 over the right hemisphere in control readers, indicating that greater responsiveness in the right OT cortex to words led to faster reading speeds (measured in syllables per second). This specific data directly associates the presence of an orthographic region in the right hemisphere with enhanced reading abilities.

Overall, it is suggested that the acquisition of music literacy could aid in improving reading skills in children who are at genetic risk for dyslexia or predisposed to it. This is due in part to the development of a bilateral orthographic area. Additionally, the intensive training in attentional and ocular displacement, which involves V5 and the oculomotor area, may serve as an enhancing and protective factor for reading ability^33,112^.

Given the insufficient activation of the left VWFA in surface dyslexic readers^90^, the functionality of a right homologous area for processing orthographic information would be a valuable neural aid. In addition, a possibly anomalous right-sided lateralization of phonological functions (described in children at genetic risk for dyslexia^113,114^) would benefit from the functionality on a right intra-hemispheric grapheme-to-phoneme conversion circuit. In both cases, music literacy might serve as a protective or rehabilitating factor for reading disorders.

It is worth noting that all study participants were fully right-handed. Therefore, functional hemispheric asymmetries cannot be attributed to individual differences but rather to their intensive music reading training (in comparison to a lack thereof). It was statistically shown that musicians' "word" training, resulting from book reading, was not as pronounced as that of control students.

The study clearly shows how (comparatively) poor readers did not show activation of the right OT cortex during word processing. A similar disadvantage was reported in face processing for unilateral versus bilateral cerebral activation, whereby males exhibited activation only in the right face fusiform area or FFA, leading to poorer performance than females in facial expression recognition tasks. This was reported by Proverbio^115^. Again, Koshik et al.^116^ reported that during tridimensional rotation of shapes, females exhibited activation in the right parietal cortex but not the left, which was found to correlate with poorer performance in mental rotation tasks.

Further investigations should be carried out on samples of adult dyslexic patients (musicians vs. non-musicians), although it would be complicated to disentangle the effects of compensating factors (neural plasticity) from the genetically inherited atipicality in neural wiring.

## Data Availability

The authors confirm that the data supporting the findings of this study are available within the article. Other information are available on request from the corresponding author. The data are not publicly available due to privacy or ethical restrictions.
